# Primary breast angiosarcoma: a rare presentation of rare tumor – case report

**DOI:** 10.1186/s12907-017-0055-y

**Published:** 2017-08-29

**Authors:** Fayçal Abbad, Najat Cherif Idrissi, Btissam Fatih, Bouchra Fakhir, Jamal Drissi, Mouna Khouchani, Hanane Rais

**Affiliations:** 1 Pathology department – Ar Razi Hospital, UCH Mohammed VI, Marrakech, Morocco; 2 Radiology department – Ar Razi Hospital, UCH Mohammed VI, Marrakech, Morocco; 3 Gyneco-obstetric surgery department, Mother-Child Hospital, UCH Mohammed VI, Marrakech, Morocco; 4 Oncology and radiotherapy department – Oncology and haematology center, CHU Mohammed VI, Marrakech, Morocco

**Keywords:** Primary angiosarcoma, Breast, Immunoprofile

## Abstract

**Background:**

Primary breast angiosarcoma is defined as malignant proliferation showing endothelial differentiation. It is a very rare tumour (0.05% of primary mammary cancers), whose diagnosis can be difficult.

**Case presentation:**

We report the observation of a patient with no previous history, aged 27 years. The clinical examination finds a right breast discreetly increased in volume. The trucut biopsy was in favour of a lactating tubular adenoma. However, an immunohistochemical complement was requested. An absence of pancytokeratin labelling contrasted with strong expression of CD31, CD34 (endothelial markers) are described. The proliferation index (Ki67) was estimated at 30%. This led to the conclusion that the phenotypic aspect is related to a vascular proliferation that evokes an angiosarcoma. After a multidisciplinary assessment, the patient benefited from an enlarged excision of the tumour. The histopathological examination of the surgical specimen found an infiltrating mesenchymal proliferation made of vessels of variable sizes anastomosed to vascular slits with lesional limits. The immunohistochemical examination on the surgical specimen showed to the same phenotypic profile on biopsy. The final diagnosis was a high-grade mammary angiosarcoma of incomplete excision. The patient refused any additional surgical management; external radiotherapy and close supervision were prescribed. After eight months of evolution, no local or remote recurrence was reported.

**Conclusion:**

Primary breast angiosarcoma is a mesenchymal malignant tumour of rare vascular origin. Our observation is peculiar by the absence of any prior radiotherapy, its clinical presentation, its morpho-phenotypic characteristics, its management and its evolutive aspects.

## Background

Primary breast sarcomas are rare entities. These malignant tumours originate from mesenchymal breast tissue and account for less than 1% of all breast cancer cases. Angiosarcomas are rare malignant tumours that arise from endothelial cells lining vascular vessels. Most angiosarcomas are known to be induced by radiation. Primary angiosarcomas are rare and account for 0.05% of all malignant breast tumours.

## Case presentation

We report the case of a 27-year-old female, with no history of previous breast surgery or irradiation. She reported after four months of breastfeeding a history of painless progressive lump in her left breast.

**Fig. 1:**
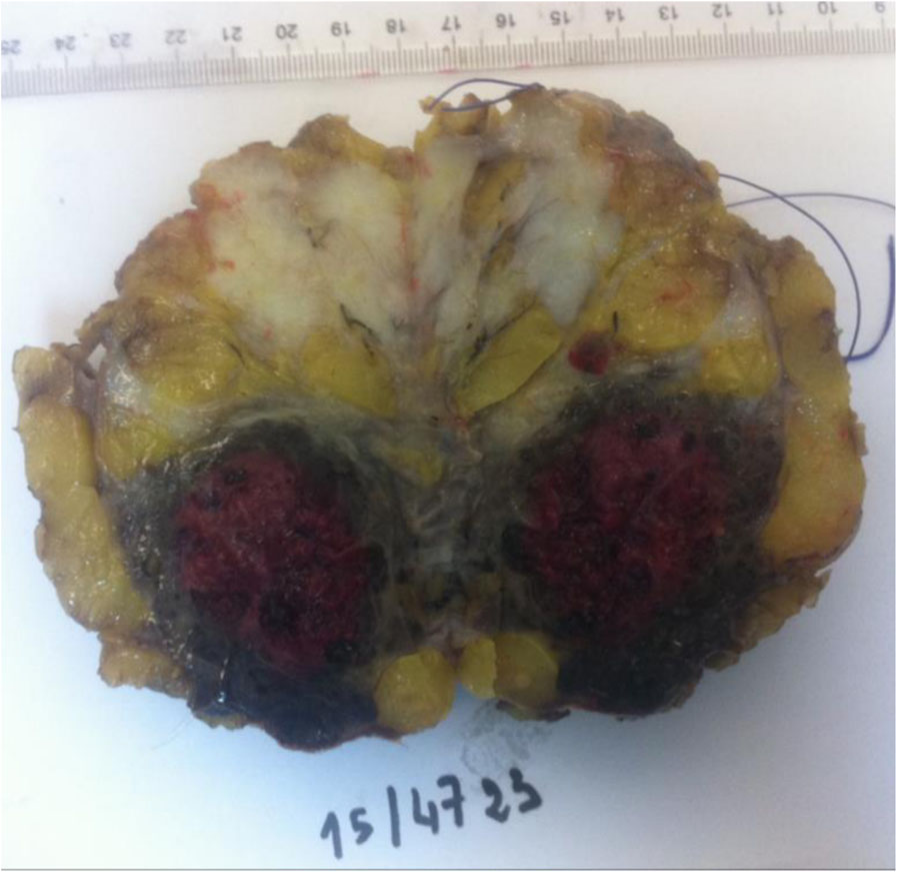
Gross examination: cut off surface of angiosarcoma lumpectomy

**Fig. 2:**
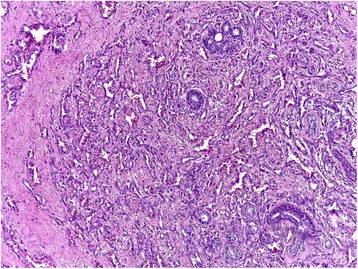
Micrography showing vascular proliferation dissecting the mesenchymal breast tissue (×10)

**Fig. 3:**
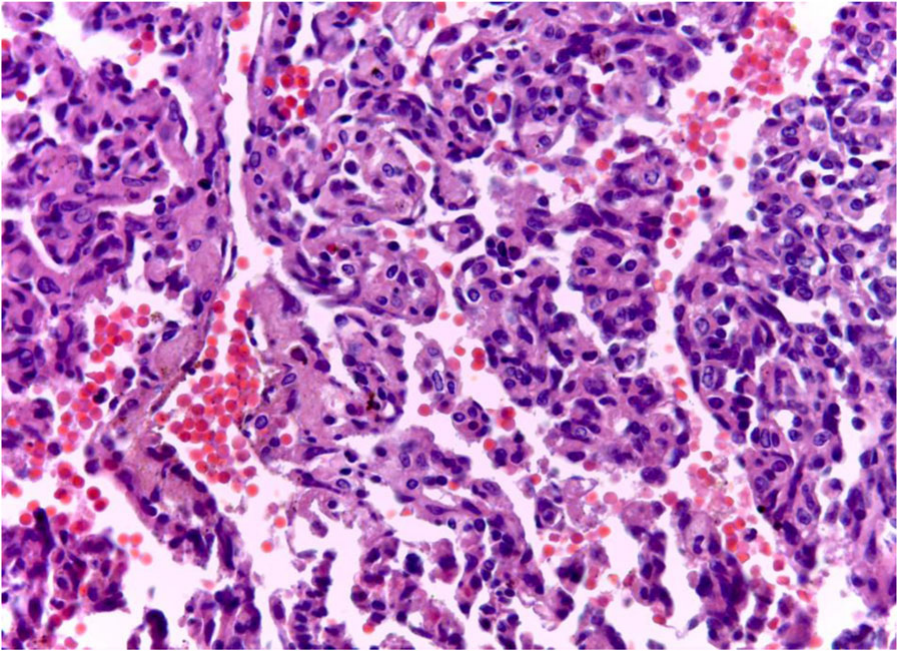
Micrography showing a papillary pattern of the angiosarcoma with high nuclear pleomorphism

**Fig. 4:**
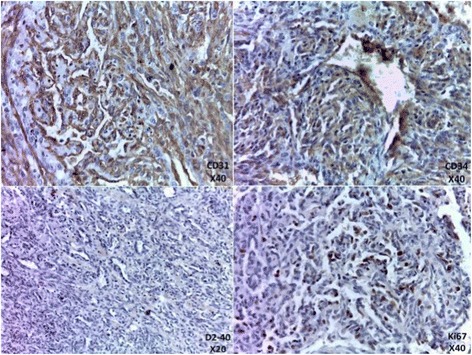
Immunohistochemical profile of our case

On examination the patient was in good condition. The right breast was slightly increased in volume. We found a 5 cm lump involving all quadrants of the breast with no cutaneous involvement. Ganglionic area and axilla were free. The sonography showed a hypo echoic (Doppler vascularized) nodular formation with fuzzy limits. The mammography revealed a homogeneous opacity, dense, with poorly defined contours, on the two internal quadrants with a retromammelonal contingent (labelled ACR-3). The trucut biopsy was in favour of a lactating tubular adenoma. The radioclinic discordance, the morphological aspect and the exiguous nature of the material, an immunohistochemical complement was requested. An absence of pancytokeratin labelling contrasted with strong expression of CD31, CD34 (endothelial markers) is described. The proliferation index (Ki67) was estimated at 30%. This led to the conclusion that the phenotypic aspect is related to a vascular proliferation that evokes an angiosarcoma. After a multidisciplinary reassessment, the patient benefited from a conservative treatment by an enlarged excision of the tumour (Fig. 1). The histopathological examination of the surgical specimen found an infiltrating mesenchymal proliferation made of vessels of variable sizes anastomosed to vascular slits with lesional limits (Fig. 2). The endothelial cells were often spindle shaped, with anisocaryotic nuclei and numerous mitoses and reduced cytoplasm (Fig. 3). The immunohistochemical examination on the surgical specimen showed to the same phenotypic profile on biopsy (Fig. 4). The final diagnosis was a high-grade mammary angiosarcoma of incomplete excision.

The patient refused any additional surgical management or external radiotherapy and close supervision were prescribed. After eight months of evolution, no local or remote recurrence was reported.

## Discussion

Breast angiosarcoma is defined as malignant proliferation showing endothelial differentiation [1]. It is divided into two distinct groups. Primary, which arises in the breast parenchyma and secondary, which develops in the skin, chest wall or breast parenchyma subsequent to surgery and postoperative radiation for breast cancer.

Mammary angiosarcomas, both primary and secondary, may show mutations in the receptor of tyrosine kinase gene KDR and high levels of Myc amplification [1–3].

Primary angiosarcoma has an incidence of about 0,05% of all primary malignancy in the breast. It is more frequent in young women (20 to 50 years) with no previous cancer history or other known risk factors [3, 4]. Up to 12% of primary breast angiosarcoma are diagnosed during pregnancy or shortly after suggesting hormonal involvement. However, oestrogen and progesterone receptors were reported to be negative in most cases [5]. The rapid growth of the disease during pregnancy and lactation are thought to be related to the suppressed immune system and placental growth factors, besides hormonal effects.

Patients with primary angiosarcoma present with a palpable mass that may be growing rapidly as seen in our case. Skin involvement is frequent (bluish red discoloration, haemorrhage). Distant metastasis can be found. The mean tumour size of the mass at presentation vary from 1 to 25 cm (average 5 cm), while in this case patient presented with a larger lump of size 17× 14 × 7 cm. Mammographic characteristics can establish the diagnostic, but frequently, as in our case, it is non specific. Sonography and magnetic resonance imaging are more sensitive in characterizing those breast lesions, but again there are no distinctive features of angiosarcomas [4, 6–9].

Diagnosis prior to surgery, either by fine needle aspiration or needle core biopsy is difficult. Authors have reported a false negative rate of 37% [3, 9]. Surgical excision and sufficient sample for histopathological examination with immunochemistry are necessary to render a final diagnosis.

Morphologically, there is a broad spectrum of growth patterns and nuclear atypia. Well-differentiated angiosarcomas consist of anastomosing vascular channels that dissect through adipose tissue and lobular stroma. Other architectural patterns include vasoformative growth, solid growth, papillary endothelial growth, and capillary-type pattern. Tumour cell shape may be typical endothelial shape, plump, spindled, or epithelioid. Nuclear atypia and mitoses may range from none to severe and numerous. Blood lakes and necrosis may be prominent. There is no possible morphological distinction between primary and secondary breast angiosarcoma. The immunophenotype will prove the endothelial differentiation. Immunohistochemical staining for CD31, CD34 or sometimes Podoplanin (D2–40) is very useful in poorly differentiated tumours. However, progressive tumour dedifferentiation can lead to a loss of those markers [9–13].

Three groups of breast angiosarcoma were proposed by Donnel et al. [12]: well differentiated, Intermediate-grade and low-grade angiosarcoma. It is based on the constellation of growth patterns, atypia, and mitotic activity.

The histological grading was thought to be predictive of the prognosis. Recent data, suggest that in angiosarcoma grade has no prognostic value. Low-grade lesions can metastasize. Second locations occur in the lungs, liver, bone and skin. Involvement of axillary lymph nodes is rare [13–16].

The differential diagnosis is variable according to this grade. It includes benign haemangioma, angiomyolipoma, melanoma, undifferentiated carcinoma, stromal sarcoma and reactive spindle cell proliferative lesion [17, 18].

The management of breast angiosarcoma is based on large surgical excision. Total mastectomy is the rule. Haematogenous dissemination of the angiosarcoma is making axillary lymph node dissection unnecessary. In high-grade angiosarcoma, chemotherapy showed a better outcome (cyclophosphamide, anthracyclin, or an alkylating agent combined with a pyrimidine analogue). In case of local recurrence, radiation therapy might be indicated. There is no clear agreement on pre-operative radiotherapy in the metastatic setting [15–17].

Most authors, link the outcome to the tumour size at diagnosis, and margin status at surgery. Median recurrence free survival is inferior to 3 years. Five year overall survival is 46% for primary breast angiosarcoma and 69% for secondary angiosarcoma [19–21].

## Conclusion

Breast angiosarcomas are rare tumours. In young women, tumours with highly vascular component at the biopsy should be considered malignant until proven otherwise. The therapeutic outcome and the prognosis are determined by tumour size, margin status and secondary location.
